# Ultrasound-mediated delivery of brain-penetrating nanoparticles across the blood-tumor barrier

**DOI:** 10.1186/2050-5736-3-S1-P34

**Published:** 2015-06-30

**Authors:** Kelsie Timbie, Clark Zhang, Elizabeth Nance, Ji Song, Wilson Miller, Justin Hanes, Richard Price

**Affiliations:** 1 University of Virginia, Charlottesville, Virginia, United States; 2 Johns Hopkins University, Baltimore, Maryland, United States

## Background/introduction

The intact blood-brain barrier (BBB) presents a major obstacle for drug delivery to the brain. In addition, both high interstitial pressure and a nanoporous electrostatically charged tissue composition, produce a “blood-tumor barrier” (BTB), further complicating the treatment of diseases like glioblastoma. Focused ultrasound (FUS) in conjunction with microbubbles (MB) has been shown to cause reversible, localized disruption of the BBB. Incorporating MR guidance with FUS offers the ability to exquisitely target the BBB disruption to specific regions of the brain, thereby permitting drug delivery in a highly localized manner. This work examines the ability of MR guided FUS to deliver highly specialized brain-penetrating nanoparticles (NP) across both the BBB and the BTB in tumor-bearing rats. NPs were 60 nm in diameter and covered with an exceptionally dense brush layer of PEG to permit excellent diffusion through brain tissue. Initial studies utilized fluorescent polystyrene tracer particles to measure NP delivery and inform dosing of cisplatin-loaded biodegradable NPs.

## Methods

One to two weeks prior to FUS treatment, 160-170 g rats were inoculated intracranially with luciferase-transfected 9L cells. On the day of treatment, the heads of the anesthetized rats were depilated and positioned in a degassed water bath coupled to the FUS system. Rats received an intravenous co-injection of NPs and MBs 30 seconds before sonication. All sonications were performed using a 1.14 MHz single element focused transducer operating at a 0.5% duty cycle for 2 minutes. Peak negative pressure was 0.6 MPa. High resolution contrast-enhanced MR images were utilized to visualize the tumor region and place sonication focal points with high accuracy. Targets were chosen to cover the entire tumor region as well as the immediate tumor periphery, thereby disrupting both the BTB and the intact BBB. Immediately following sonication, MRI contrast agent was delivered intravenously and T1-weighted contrast enhanced MRI images were captured to verify BBB disruption. Animals were recovered and monitored for 1hr-2 weeks post treatment. In animals receiving fluorescent tracer NPs, brains were perfused with 2% heparinized saline, dessicated and cryosectioned. Mounted sections were stained with BS-I lectin to reveal endothelial cells (ECs) and imaged with fluorescent microscopy. In animals receiving drug-loaded NPs, tumor growth post treatment was measured using IVIS.

## Results and conclusions

**Figure 1:**
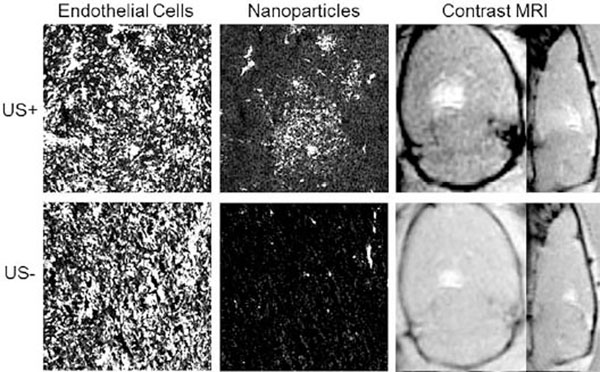
FUS and MBs are able to deliver 60 nm NPs across the BTB. Contrast-enhanced MR images demonstrate FUS-mediated BBB opening in the tumor periphery, compared to US-images. Fluorescent images show clear enhancement of NP delivery in FUS+ tumors

Initial studies with fluorescent tracer particles indicate that FUS and MBs are capable of disrupting the BTB and enhancing the delivery of 60 nm NPs in the tumor region. In Figure 1, fluorescent microscopy shows significant NP delivery in a FUS+ tumor, equating to a 30-fold increase when compared to a FUS-tumor. Although the BBB is compromised in the tumor region, as indicated by pre-sonication MR imaging, additional treatment with FUS and MBs was required to overcome the BTB and permit significant NP delivery. Combined with prior work demonstrating the ability of FUS to deliver these large NPs to healthy brain tissue, our results provide promising evidence that delivery of a therapeutic drug dose to both the tumor core and periphery is possible, and work continues in this area.

